# Treatment strategies for postoperative locoregional recurrent esophageal squamous cell carcinoma after curative resection: a narrative review

**DOI:** 10.1007/s10388-026-01234-y

**Published:** 2026-07-20

**Authors:** Keiichi Jingu, Rei Umezawa, Takaya Yamamoto, Noriyoshi Takahashi, Ken Harada, Yu Suzuki, Keita Kishida, So Omata, Hinako Harada, Nanae Chiba, Shinsaku Okuda, Sota Uchimi, Masato Hirano, Yushi Takahashi, Kouki Mori

**Affiliations:** Department of Radiation Oncology, Tohoku University Graduate School of Medicine, 1-1 Seiryo-chou, Aoba-ku, Sendai, 980-8574 Japan

**Keywords:** Recurrent esophageal cancer, Chemoradiotherapy, Oligo-recurrence, Locoregional recurrence, Radiotherapy, Immunotherapy

## Abstract

Recurrence following curative esophagectomy remains a major clinical challenge and is associated with poor prognosis. Treatment strategies for postoperative recurrent esophageal squamous cell carcinoma must be individualized based on recurrence patterns, prior treatment history, and patient-related factors. Locoregional recurrence and oligo-recurrence in some non-regional lymph nodes may still be amenable to curative-intent therapy, including salvage surgery and definitive chemoradiotherapy (CRT). Selection of optimal treatment should consider tumor burden, performance status, and prior radiation exposure. Salvage surgery offers favorable long-term outcomes in carefully selected patients, particularly when recurrence occurs in non-dissected regions. CRT remains a central treatment strategy for unresectable disease, whereas re-irradiation after prior radiotherapy carries a substantial risk of severe toxicity and requires cautious patient selection. Advances in radiotherapy techniques, including intensity-modulated radiotherapy and particle therapy, may improve therapeutic efficacy while reducing toxicity. In parallel, systemic therapies, particularly immune checkpoint inhibitors, have expanded treatment options; however, their role in locoregional recurrence remains to be fully defined. Importantly, prognostic factors should not only be considered descriptive variables but also integrated into clinical decision-making to distinguish candidates for curative versus palliative treatment. Future strategies integrating precision oncology and multimodal approaches are expected to further improve outcomes. Prospective studies are needed to establish optimal treatment algorithms.

## Introduction

Esophageal squamous cell carcinoma is associated with high rates of morbidity and mortality. Despite significant advances in multimodal treatments, including surgery, chemoradiotherapy (CRT), and systemic chemotherapy, it is often diagnosed at advanced stages and exhibits a poor prognosis. In Japan, approximately 37%–43% of patients with esophageal cancer experience recurrence after radical surgery [1, 2]. Recurrence commonly occurs in the lymph nodes, lungs, liver, and bone or in a combination of these sites. However, in recent years, the recurrence pattern has changed due to the widespread adoption of preoperative treatment. Among patients with recurrence enrolled in the JCOG1109 study, 55% presented with distant recurrence, 28% with locoregional recurrence, and 17% with both patterns [3].

Recurrence after curative surgical resection is common and significantly worsens survival outcomes. Therefore, precisely identifying the type of recurrence, i.e., locoregional, oligo-recurrence in nonregional lymph nodes, oligo-recurrence as distant visceral metastases, or widespread distant metastases, is crucial for selecting the optimal treatment strategy. Recent advances in therapeutic modalities, especially in radiotherapy (RT) techniques and systemic therapies, offer new strategies for managing recurrent esophageal cancer. While treatment strategies may vary depending on the histological subtype, this review focuses on squamous cell carcinoma, one of the major histological subtypes of esophageal cancer. This is because there is insufficient data on salvage therapy for adenocarcinoma, another major histological subtype. This review aims to provide a comprehensive and clinically oriented overview of current treatment strategies, integrating recent evidence and emerging concepts.

## Recurrence classification

Recurrence after curative esophagectomy can be broadly categorized into four patterns:

1. Locoregional recurrence (tumor bed, anastomotic site, and regional lymph nodes).
2. Oligo-recurrence in nonregional lymph nodes (≤ 5 nodes).
3. Oligo-recurrence in distant organs (liver and lung).
4. Widespread metastatic disease.

Decisions are guided by several key factors: number and location of lesions, histology, disease-free interval (DFI), patient’s performance status, comorbidities, prior treatments, and feasibility of salvage surgery or RT. Oligo-recurrence was defined by Niibe as a condition in which patients have ≤ 5 metastatic or recurrent lesions with controlled primary tumors [4]. Locoregional recurrence typically affects the original tumor bed, anastomotic site, or adjacent lymph nodes. In general, survival rates are more favorable for locoregional recurrence than for distant recurrence [5, 6]. The distinction between regional and nonregional lymph node recurrence remains controversial. Nonregional lymph node metastases may reflect systemic disease rather than true locoregional recurrence, and their biological behavior may differ substantially. However, some nonregional lymph node metastases, such as supraclavicular, pretracheal, tracheobronchial, and posterior thoracic paraaortic nodes, may still be amenable to curative local therapy. In contrast, other sites, including abdominal paraaortic or posterior pancreatic head lymph nodes, are more likely to represent systemic disease. Therefore, nonregional lymph node recurrence should not be uniformly classified as locoregional disease or widespread metastatic disease, and treatment decisions should be individualized based on anatomical location, biological behavior, and potential for curative local therapy. This review will focus on locoregional recurrence and oligo-recurrence in some nonregional lymph nodes.

## Prognostic factors

Prognostic factors play a critical role in guiding treatment selection.

Among patient-related factors, baseline performance status is a consistent predictor of outcome. Multiple retrospective series, including large multi-institutional studies of nodal oligo-recurrence, have found that patients with an ECOG performance status of 0–1 or a Karnofsky performance status of ≥ 80% have significantly longer OS and PFS after salvage therapy.

Recurrence-related factors are especially influential. A longer DFI from the primary surgery to recurrence—typically defined as ≥ 12 months—is one of the most robust positive prognostic indicators [7]. Patients with a single or few recurrent lymph nodes in one area have better outcomes than those with multiple lesions. Tumor burden is also important; a smaller maximum nodal diameter, with thresholds reported between 22 and 35 mm [7–9], is associated with improved OS and PFS. The anatomical location within the locoregional area can also affect prognosis; isolated nodal failure is generally associated with a more favorable prognosis than anastomotic recurrence or recurrence involving multiple nodal basins. Notably, Jingu et al. also reported the results of CRT for isolated lymph node metastases after curative surgery, showing that the median OS for patients with supraclavicular lymph node metastases who were suitable for surgery was 117.5 months [10], although this was a small, retrospective study.

Overall, these findings highlight the importance of comprehensive baseline evaluation and individualized treatment selection for patients with postoperative locoregional recurrent esophageal cancer. The combination of favorable host factors, limited and slow-growing disease, and aggressive local therapy offers the potential for long-term survival in this challenging patient group. Importantly, these prognostic factors should not be regarded merely as descriptive variables but should be actively incorporated into clinical decision-making. Patients with favorable characteristics may be considered potential candidates for curative-intent therapy, although these observations are primarily derived from retrospective analyses and may be influenced by selection bias. On the other hand, patients with multiple adverse factors may be better managed with palliative approaches. Table 1 summarizes key prognostic factors and their clinical implications for treatment selection.

**Table 1:** Prognostic factors at each phase in locoregional recurrence

Pretreatment factors	Treatment-related factors	Posttreatment factors
Age	Local treatment	Treatment response
Disease-free interval	Surgery (preferred)	
No anastomotic recurrence	Concurrent CRT† (2nd option) Taxane + 5-FU + RT /taxane + CDDP + RT /taxane + CDDP + 5-FU + RT	
Cervical lymph node metastases	ICIs‡+RT§ (3rd option)	
Single region involvement	RT§ alone (alternative)	
Solitary lymph node	RT§ dose ≥ 60 Gy	
Tumor diameter		
Performance status		
Pathological stage		
RT§ history		

*RT§* radiotherapy, *CRT†* chemoradiotherapy, *ICIs‡* immune checkpoint inhibitors

## Treatment strategy framework

**Fig. 1:**
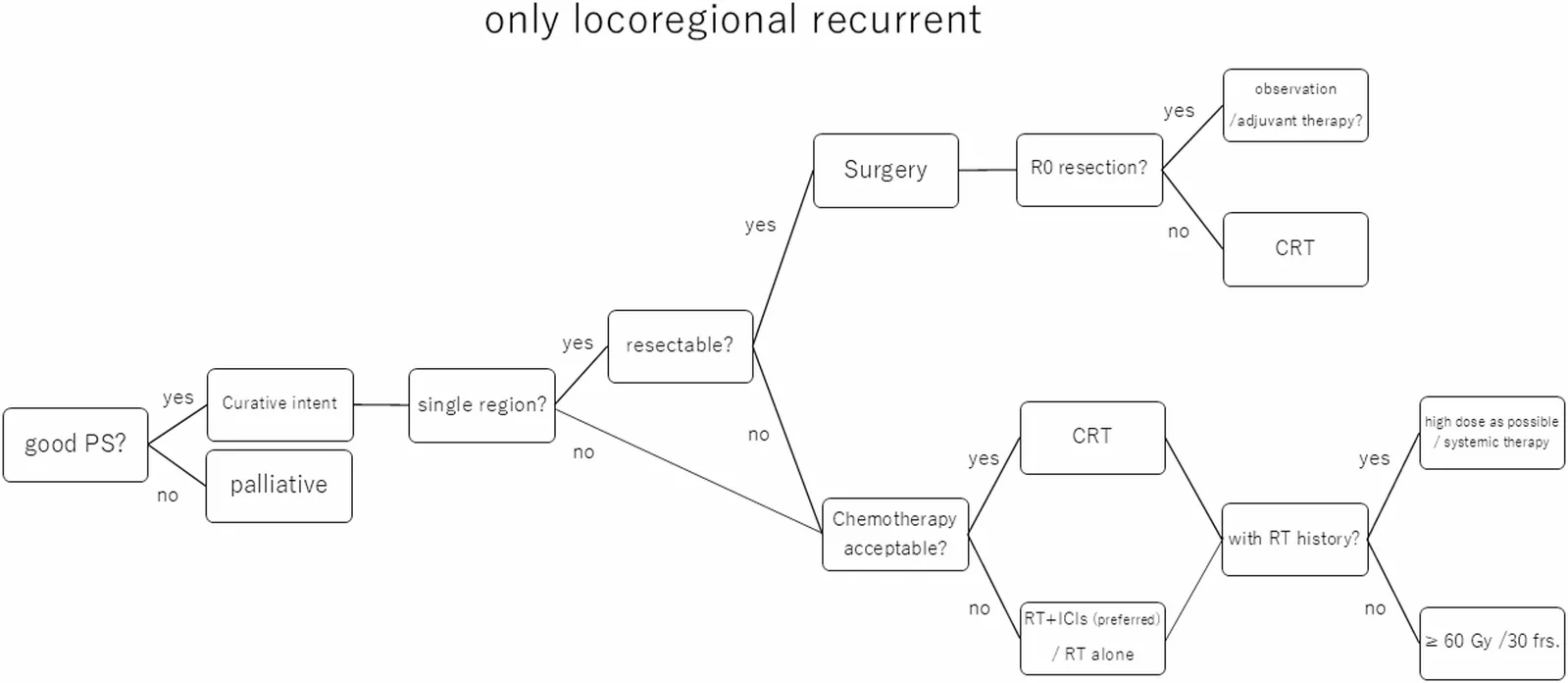
Flow chart of the treatment strategy for locoregional recurrence

Treatment strategies for postoperative locoregional recurrence should be individualized based on resectability, prior treatment history, and recurrence characteristics. Available treatment options include salvage surgery, definitive CRT, and re-irradiation (Fig. 1).

### Salvage surgery

Surgical resection, when feasible, may provide favorable long-term outcomes in selected patients. In particular, a favorable long-term prognosis is expected for cases with recurrence limited to supraclavicular lymph node metastases [11–14]. Appropriate patient selection is critical for salvage surgery. Although salvage surgery is a potentially curative treatment option, it must be approached with caution due to its high complexity and associated risks. A critical consideration is whether recurrence occurs within previously dissected regions. Recurrence in previously dissected fields is often associated with extensive fibrosis and adhesions, making surgery technically challenging and associated with high morbidity. In contrast, recurrence in non-dissected lymph node regions may be more amenable to surgical resection and associated with better outcomes [15]. Moreover, it remains unclear whether adjuvant therapy is necessary when an R0 resection is achieved.

### Chemoradiotherapy

A retrospective analysis of CRT showed better survival outcomes than RT alone or chemotherapy alone, especially when definitive radiation doses (typically ≥ 60 Gy) were administered along with systemic chemotherapy [11, 16]. Elective nodal irradiation has not demonstrated clear clinical benefit in the current literature [17–19]. Modern RT modalities such as intensity-modulated radiation therapy (IMRT), stereotactic body radiation therapy (SBRT) [16, 20], proton therapy [21], and carbon-ion therapy enable the delivery of high radiation doses to recurrent sites while sparing surrounding organs. Proton and carbon-ion therapies have yielded encouraging initial results in local control and tolerability for isolated nodal recurrence [22–24]. Analysis of recurrence patterns after CRT for postoperative locoregional recurrent esophageal cancer revealed that local control failure rates and new metastatic recurrence rates were similar [25]. This finding highlights that improving local control is as critical as preventing metastasis, suggesting that these new RT techniques may be key to a better prognosis.

Regarding concurrent chemotherapy regimens with RT, while the CDDP + 5-FU regimen is widely used, the CDDP + paclitaxel regimen has shown promising efficacy [26, 27].

### Re-irradiation

Because re-irradiation is associated with a substantial risk of severe toxicities, including gastric tube necrosis or perforation and tracheal necrosis, radiation doses are often reduced to minimize treatment-related complications [28, 29]. Re-irradiated patients demonstrated worse OS and local control than patients receiving first-time radiotherapy. On the other hand, Wu et al. demonstrated that a higher re-irradiation dose (55–60 Gy) *was associated with* better outcomes [30]. *However*,* the favorable outcomes reported by Wu et al. may also reflect patient selection and treatment limitations inherent to the re-irradiation setting.* Therefore, *re-irradiation should be reserved for highly selected patients in whom no safer curative treatment options are available.*

## Outcomes of treatments for postoperative locoregional recurrent esophageal cancer

Salvage surgery yields better long-term outcomes compared to RT or CRT, especially for cervical lymph node recurrence [12, 31]. In terms of the long-term outcomes, the median survival period after salvage surgery was longer than 24 months, consistent with the literature (Table 2).

**Table 2:** Historical outcomes of treatments for postoperative locoregional recurrent esophageal cancer

Author	Year	Study design	No.	Methods	3-year overall survival rate	Median survival period
JL Raoul [33]	1995	Retrospective	24	CRT	4.3%	10 months
Nemoto [32]	2001	Retrospective	33	RT§ (21) CRT† (12)	9% 25%	7 months 9 months
Shioyama [8]	2007	Retrospective	82	RT (52) CRT (30)	25% 33% (2-year)	7 months
Lu [34]	2010	Retrospective	73	RT (42) CRT (31)	0% 33.8%	9 months 17 months
Zhang [26]	2012	Retrospective	50	CRT	14%	13.3 months
Jingu [37]	2012	Prospective	30	CRT	38.4%	21 months
Zhang [38]	2015	Retrospective	27	CRT	60.2% (2-year)	26 months
Jeene [36]	2017	Retrospective	39	CRT	31%	22 months
Chen [35]	2020	Prospective	48	CRT	49.7%	27.4 months
Ito [9]	2022	Retrospective	51	CRT	40.7%	23.1 months

*RT§* radiotherapy alone, *CRT†* chemoradiotherapy

In the 1980s and early 1990s, RT was mainly used as a palliative measure for recurrent esophageal cancer. Radiation was typically delivered using conventional techniques (e.g., two-dimensional RT) at doses ranging from 30 Gy in 10 fractions to 50 Gy in 25 fractions. Radiotherapy alone was primarily used. The median survival time was generally under 6–8 months [32, 33]. In the late 1990s and 2000s, three-dimensional conformal RT (3D-CRT) was introduced, which improved dose conformity and enabled dose escalation up to 60 Gy [8, 26, 34]. As it became clear that treating isolated locoregional recurrence was feasible, RT started to be used with curative intent, especially in patients with limited recurrence and good performance status. In the 2010s and early 2020s, the combination of precision RT and chemotherapy (typically cisplatin + 5-FU or nedaplatin + 5-FU) significantly improved treatment efficacy. CRT became a standard of care for nonsurgical candidates with locoregional recurrence. The median survival time increased to approximately 24 months [9, 35–38].

For locoregional recurrence, new therapeutic strategies were highlighted at the 2024 ASCO Annual Meeting, where preliminary findings from a phase II trial conducted by Jingu et al. demonstrated promising efficacy of concurrent CRT with docetaxel, cisplatin, and 5-FU (DCF regimen) combined with RT [39]. The results showed a 5-year progression-free survival (PFS) rate of 56.2% and a median PFS of 62.0 months, with no patients experiencing grade 2 or higher late toxicity. This trial suggested promising improvements in PFS and OS compared with conventional CRT, with minimal toxicity. However, because this was a single-arm phase II study without a randomized comparator, the findings should be interpreted cautiously. Large-scale randomized phase III trials comparing DCF-RT with conventional CF-RT are warranted.

## Integration of precision oncology and immunotherapy

### Genomic profiling

Precision medicine strategies have been increasingly integrated into the management of recurrent esophageal cancer. Advances in genomic profiling and biomarker development have enabled more personalized treatment planning. Circulating tumor DNA (ctDNA) monitoring has emerged as a valuable tool for the early detection of recurrence, assessment of treatment efficacy, and identification of actionable mutations for precision therapy. ctDNA-guided strategies hold promise for personalizing and enhancing treatment outcomes in patients with postoperative recurrent esophageal cancer [40–42].

### Immune checkpoint inhibitors

Immunotherapy, particularly immune checkpoint inhibitors (ICIs) targeting PD-1 (e.g., nivolumab and pembrolizumab), has significantly advanced the treatment landscape. Studies have shown improved outcomes in patients with PD-L1 expression [43], microsatellite instability (MSI) [44], or a high tumor mutational burden (TMB) [45]. Combination strategies integrating immunotherapy with chemotherapy or RT have shown a synergistic potential and are under investigation.

The choice between these modalities should be based on individual patient factors, such as performance status, recurrence characteristics, biomarker status, prior treatment history, and potential toxicities. CRT remains vital for immediate symptom relief and effective local control, especially for bulky lesions causing obstruction or significant local symptoms. Immunotherapy may be a better choice for patients with smaller-volume recurrences or those with a high potential for systemic diseases.

### Combinatorial immunotherapy and CRT

Although immunotherapy combined with chemotherapy has improved outcomes in subgroups of patients with unresectable advanced or recurrent esophageal squamous cell carcinoma compared with chemotherapy alone [46], its benefit in locoregional recurrence remains uncertain. In this setting, the median overall survival (OS) was 14.8 months in the nivolumab plus chemotherapy group, 13.9 months in the nivolumab plus ipilimumab group, and 13.5 months in the chemotherapy-alone group, suggesting a limited survival benefit in this exploratory subgroup analysis. Direct comparative trials between CRT alone and CRT combined with immunotherapy for postoperative locoregional recurrent esophageal cancer are scarce. However, retrospective data suggest that the addition of local therapy is critical. Wu et al. showed that the median OS in patients receiving RT combined with immunotherapy (19.48 months) was significantly longer than that in patients receiving immunotherapy alone (7.69 months) [47]. RT may also enhance antitumor immune responses through immunomodulatory effects, including increased antigen presentation and potential induction of the abscopal effect. These mechanisms provide a biological rationale for combining CRT with ICIs in locoregional recurrent esophageal cancer. Recent prospective studies, including the TENERGY trial [48], have demonstrated the feasibility of integrating immunotherapy with definitive CRT in esophageal squamous cell carcinoma. Although evidence remains preliminary, these approaches may improve both local and systemic disease control.

Taken together, these findings suggest that effective local control remains essential even in the era of immunotherapy. Nevertheless, emerging evidence from other malignancies supports the potential of combining CRT with immunotherapy to enhance treatment efficacy. Prospective clinical trials are warranted to determine the optimal integration of CRT and immunotherapy and to establish evidence-based treatment strategies. Biomarker-driven approaches, including the use of ctDNA and MSI, are expected to further refine patient selection and optimize individualized treatment strategies.

### Management of recurrence after adjuvant immunotherapy

The introduction of adjuvant nivolumab following neoadjuvant chemoradiotherapy and surgery, based on the results of the CheckMate 577 trial [49], has significantly changed the treatment landscape for esophageal squamous cell carcinoma. As a result, clinicians are increasingly encountering locoregional recurrence in patients previously exposed to immune checkpoint inhibitors (ICIs).

However, optimal treatment strategies for postoperative locoregional recurrence after prior immunotherapy remain unclear. In particular, the efficacy of ICI rechallenge and the role of combined local and systemic therapies in this setting have not yet been established.

For patients with isolated locoregional recurrence after adjuvant immunotherapy, definitive local therapy, including salvage surgery or CRT, may still play a critical role. Because prior exposure to immune checkpoint inhibitors may reduce the effectiveness of subsequent immunotherapy in some patients, achieving durable local control could become even more important in carefully selected patients. In addition to conventional CRT, intensified CRT regimens such as DCF-RT have shown promising efficacy. However, the currently available evidence is limited to single-arm phase II studies. Further randomized studies comparing intensified CRT approaches with conventional regimens are needed before these strategies can be widely adopted as standard treatment.

### Future directions

Emerging therapeutic strategies are reshaping the treatment landscape. These include ctDNA-guided surveillance and biomarker-driven treatment adaptation, which enable earlier detection of molecular relapse before it is visible on imaging [50]. Immunotherapy combinations, such as ICIs with chemotherapy, antiangiogenic agents, or novel costimulatory antibodies, are being studied to improve systemic control. Furthermore, the role of molecularly targeted agents targeting Epidermal Growth Factor Receptor (EGFR), Fibroblast Growth Factor Receptor (FGFR), Phosphoinositide 3-kinase (PI3K), or Cyclin-Dependent Kinase 4 and 6 (CDK4/6), is being actively investigated for biomarker-selected patient groups [51]. In patients with a high risk of distant metastasis or residual disease based on clinical and biological factors, a combination of these new systemic therapies and local treatments may be necessary.

## Suggested treatment algorithm

The management of postoperative recurrent esophageal squamous cell carcinoma should begin with comprehensive restaging to accurately define the extent and pattern of recurrence. Contrast-enhanced computed tomography (CT), positron emission tomography-computed tomography (PET-CT), and, where appropriate, magnetic resonance imaging (MRI) are essential for distinguishing locoregional recurrence from distant metastatic disease. This distinction is critical because treatment goals may differ substantially between curative-intent and palliative approaches.

For patients with isolated locoregional recurrence and good performance status, definitive local therapy should be considered whenever feasible. Salvage surgery may be appropriate in carefully selected patients, particularly when recurrence occurs in non-dissected lymph node regions and complete resection is technically achievable. However, because the available evidence is primarily retrospective and may be affected by selection bias, careful multidisciplinary evaluation is essential.

Definitive CRT remains a central treatment strategy for unresectable locoregional recurrence. Concurrent platinum-based doublet chemotherapy is commonly used, while taxane-containing regimens have shown promising efficacy in retrospective studies. In patients with prior thoracic radiotherapy, the risks of re-irradiation, including severe toxicities such as gastric conduit or tracheal necrosis, should be carefully considered. Re-irradiation should therefore be reserved for highly selected patients in whom safer alternative treatment options are not available.

For patients with poor performance status, extensive disease burden, or high risk of systemic progression, systemic therapy and palliative approaches may be more appropriate. Immune checkpoint inhibitors may be considered in selected patients, particularly in biomarker-positive populations; however, their role in locoregional recurrence remains to be fully established.

Importantly, prognostic factors including performance status, disease-free interval, tumor burden, recurrence pattern, and prior treatment history should be incorporated into treatment decision-making. Multidisciplinary assessment integrating surgical, radiation oncology, and medical oncology perspectives is essential for optimizing individualized treatment strategies.

The proposed treatment algorithm is summarized in Fig. 1.

## Conclusions and future outlook

Postoperative locoregional recurrent esophageal cancer remains a complex clinical challenge that demands careful multidisciplinary assessment and personalized treatment plans. While salvage CRT remains a central treatment strategy for nonsurgical candidates with unresectable locoregional recurrence, advances in surgical techniques, RT modalities, and systemic therapies are broadening the range of potentially curative options.

Modern conformal RT offers improved dose distribution and organ sparing, which allows for dose escalation with potentially less toxicity. Immunotherapy, especially PD-1 inhibitors, offers systemic disease control and is most effective in biomarker-selected populations. However, for locoregional recurrence, both local control and prevention of new metastases are critically important, and combined-modality approaches that integrate CRT with immunotherapy require further study.

Precision oncology tools, such as genomic profiling and ctDNA monitoring, offer the potential for earlier detection, more refined risk stratification, and customized therapy based on molecular characteristics. Integrating these methods into clinical practice can improve outcomes and minimize unnecessary toxicity.

Finally, individualized, multifaceted treatment strategies are essential for the optimal management of locally recurrent esophageal cancer after surgery. A sophisticated, risk-based treatment strategy integrating prognostic factors, past treatment history, and new systemic therapies is crucial for optimizing treatment outcomes in this challenging clinical situation. Future prospective studies incorporating biomarker-driven strategies and personalized treatment approaches are needed to establish evidence-based management for this heterogeneous disease.
